# Gene transcription changes in a locust model of noise-induced deafness

**DOI:** 10.1152/jn.00119.2021

**Published:** 2021-05-05

**Authors:** Andrew S. French, Ben Warren

**Affiliations:** ^1^Department of Physiology and Biophysics, Dalhousie University, Halifax, Nova Scotia, Canada; ^2^Department of Neuroscience, Psychology and Behavior, University of Leicester, Leicester, United Kingdom

**Keywords:** auditory neurons, hearing, mechanotransduction, noise-induced hearing loss

## Abstract

Locusts have auditory structures called Müller’s organs attached to tympanic membranes on either side of the abdomen. We measured the normalized abundances of 500 different mRNA transcripts in 320 Müller’s organs obtained from 160 locusts (*Schistocerca gregaria*) that had been subjected to a loud continuous 3-kHz tone for 24 h. Abundance ratios were then measured relative to transcripts from 360 control organs. A histogram of the number of observed transcripts versus their abundance ratios (noise exposed/control) was well fitted by a Cauchy distribution with median value near one. Transcripts below 5% and above 95% of the cumulative distribution function of the fitted Cauchy distribution were selected as putatively different from the expected values of an untreated preparation. This yielded eight transcripts with ratios increased by noise exposure (ratios 1.689–3.038) and 18 transcripts with reduced ratios (0.069–0.457). Most of the transcripts with increased abundance represented genes responsible for cuticular construction, suggesting extensive remodeling of some or all the cuticular components of the auditory structure, whereas the reduced abundance transcripts were mostly involved in lipid and protein storage and metabolism, suggesting a profound reduction in metabolic activity in response to the overstimulation.

**NEW & NOTEWORTHY** Locust ears have functional and genetic similarities to human ears, including loss of hearing from age or noise exposure. We measured transcript abundances in transcriptomes of noise-exposed and control locust ears. The data indicate remodeling of the ear tympanum and profound reductions in metabolism that may explain reduced sound transduction. These findings advance our understanding of this useful model and suggest further experiments to elucidate mechanisms that ears use to cope with excessive stimulation.

## Graphical abstract

**Figure:**
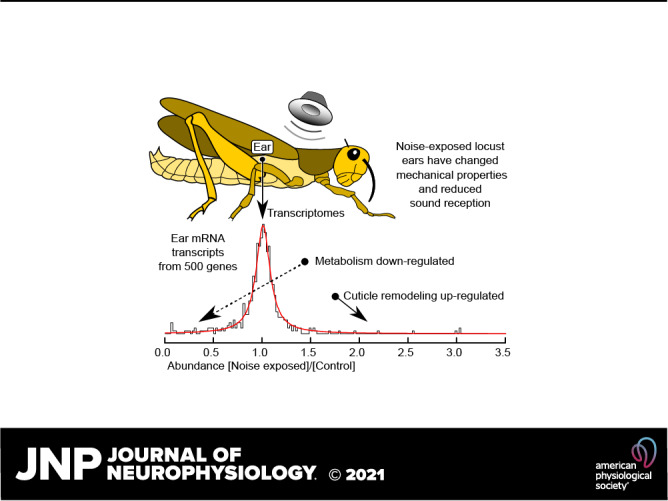

## INTRODUCTION 

About 1.5 billion people globally have compromised hearing (World Health Organization, http://www.who.int/) due to a range of causes, including genetic defects, infectious diseases, loud noise exposure, and aging. Experimental noise exposure has provided important models of deafness in mammals (1), and this has recently been extended to insect auditory systems (2, 3). Despite obvious differences, insects have evolved organs of hearing that deal with the same problems in converting the small pressure differences of sound into receptor currents in sensory neurons (4). Important similarities with vertebrates are the use of ciliated sensory neurons that use mechanical feedback to amplify small displacements (5, 6) and the presence of many homologous genes in the development and physiology of the hearing structures (7). Insects can provide experimental advantages because of their relatively simple anatomy, ease of breeding, rapid development, and reduced costs. Insects have also provided useful models of aging, including loss of hearing with age (7, 8), with the additional advantage of a short lifespan.

Audition, like all mechanoreception, can be considered as a three-stage process (9). First, the external stimulus (sound) is mechanically coupled to a sensory structure; second, the mechanical signal is transduced to cause an electrical receptor current; third the receptor current is encoded in action potentials for distance transmission. Deafness could involve malfunction at any of the three stages. Changes in mechanical properties and reductions in transduced receptor potentials have been seen in aged (7) and noise-exposed (2, 3) insects, but the causes of these changes remain enigmatic.

A range of ion channel families have been implicated in sensory mechanotransduction, including audition. These comprise Piezo proteins (10), transient receptor potential (TRP) channels (11, 12), degenerin/epithelial sodium channel/acid sensing ion channel (DEG/EnaC/ASIC) families (13), and transmembrane channel-like (TMC) proteins (14). Insect audition is performed by chordotonal organs (15), and three types of TRP channel genes have been linked to chordotonal mechanically activated ion currents: NompC (16), Nanchung, and Inactive (17, 18). However, all the above families must be considered when searching for changes in auditory function.

Many arthropod mechanoreceptors, including chordotonal sensilla, rely on transepithelial gradients of ionic concentrations and voltages to drive the receptor current through mechanically activated ion channels (19). The detailed arrangement of ionic pumps, exchangers, and channels that produce these gradients are not completely understood in any insect tissue (20), but changes in these components could clearly reduce the receptor current and sound detection.

Desert locusts, *Schistocerca gregaria*, have paired abdominal auditory organs, each consisting of an external tympanum with a sensory structure called Müller’s organ attached internally (21–23). We found previously that 24-h noise exposure produced hearing loss characterized by both mechanical and electrophysiological changes in the locust system (3). Here, we compared the abundances of 500 different mRNA transcripts from Müller’s organs in noise-exposed versus control locusts, in attempts to identify the major molecular changes underway in this model of noise-induced hearing loss.

## MATERIALS AND METHODS

### Animals, Noise Exposure, and Tissue Extraction

Details of the animal handling, noise exposure, and transcriptome creation have been given before (3). Briefly, locusts (*Schistocerca gregaria*) were reared in the gregarious phase with a 12-h light/dark cycle at 36.25°C, fed on a combination of fresh wheat and bran ad libitum. Male locusts between 10 and 20 d postimaginal molt were used for experiments. Wings were cut off at their base to increase noise exposure to the tympanal ears. Up to 20 locusts at a time were placed in a cylindrical wire mesh cage (8 cm diameter, 11 cm height) directly below a loudspeaker (Visaton FR 10 HM 4 OHM, RS Components) driven by a function generator (Thurlby Thandar Instruments TG550, RS Components) and an audio amplifier (Monacor PA-702, Insight Direct) to produce a 3-kHz tone at 126 dB sound pressure level (SPL), measured at the top of the cage, for 24 h continuously. Control locusts were selected, housed, and treated identically for 24 h, but without activating the 3-kHz tone.

**Figure 1.**
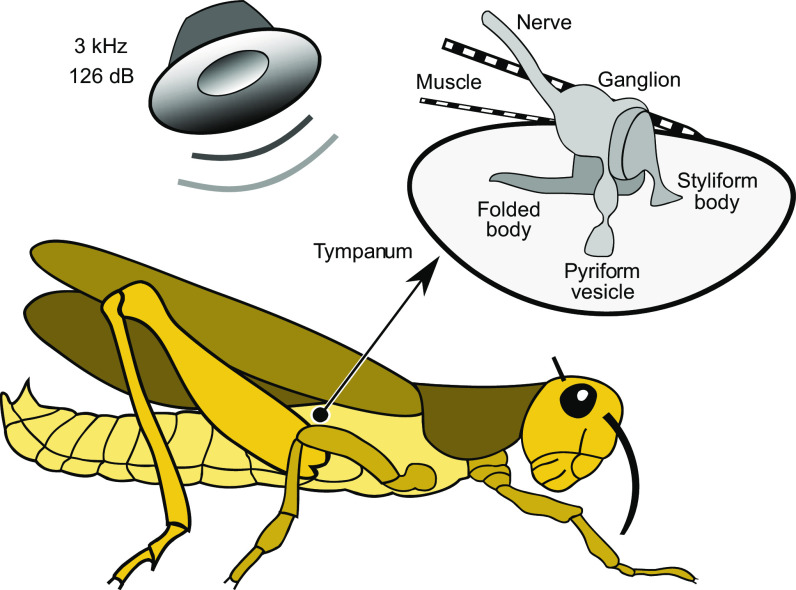
Stimulation of locust ears. Noise-exposed locusts (160 animals) were placed in a cylindrical wire mesh cage directly below a loudspeaker producing a 3-kHz tone at 126 dB sound pressure level (SPL) for 24 h continuously. Other conditions were normal (12-h light/dark cycle, 36.25°C). Controls (160 animals) were treated identically, except that the loudspeaker was silent. Locusts’ ears (black circle) comprise tympani on either the side of the abdomen, each innervated internally by a Müller’s organ, being a nerve ganglion containing at least four identifiable groups of scolopidial sensory neurons that proceed distally through the styliform, folded and pyriform structures to form close apposition with the tympanum (22, 23). At least two muscles are connected to the edge of the tympanum, close to an adjacent spiracle (not shown).

A total of 320 Müller’s organs from 160 noise-exposed locusts (2 ears per locust) were extracted by grasping the Müller’s organ (Fig. 1) through the tympanum with fine forceps and pulling it out. Another 320 Müller’s organs were extracted similarly from control animals. RNA extraction took place less than 4 h from the end of the 24-h noise exposure. Müller’s organs were snap frozen onto a pestle within an Eppendorf tube submerged in liquid nitrogen and RNA extracted and treated with DNase using an RNAqueous kit (AM1931, ThermoFisher). RNA was shipped in dry ice for Illumina HiSeq 2000 sequencing by Beijing Genomics Institute (Hong Kong). Sample RNA integrity values of 8.7 and 8.4 were given by control and noise-exposed samples, respectively. Both noise-exposed and control groups gave ∼186.1 million paired end reads of 100 nucleotides each.

### Transcript Discovery

Initial cDNA reads were groomed to select those with 80 or more contiguous nucleotides with Phred quality score of >19 to give a final database of ∼100 million pairs of reads each from control and noise-exposed groups. Two approaches were used to select transcripts for assembly. The first method was a targeted search for genes likely to be affected by noise exposure from known physiology. These included mechanically activated ion channels, membrane transporters for ions hypothetically involved in sensory transduction, cytoskeletal proteins, and molecules associated with synaptic transmission. Sequences of interest were identified by searching all possible translations of reads from the control transcriptome versus amino acid sequences of published genes using BLOSUM matching matrices (24). Closely related species were used when possible, but *Drosophila melanogaster* sequences were also used in some cases. The searches were conducted at relatively low stringency so that many unrelated genes were also found, assembled, and included in the list. Transcripts from this targeted approach included the genes that we described previously (3).

The second method attempted to find individual reads with strongly different abundances in the two transcriptomes. The first 10 million pairs of each transcriptome were searched by counting the number of times that each read was repeated identically. This process was accelerated by removing all copies of each read from the abbreviated transcriptome as it was counted. This continued until all different reads were found in each set. This initial count took ∼3 mo of continuous process by two desktop computers. A second program then searched the two lists of reads for identically matching noise-exposed and control reads in each set and calculated the ratio of the two counts. Finally, matching reads with abundance ratios exceeding 3:1 in either direction were used for assembly, commencing with the highest and lowest ratios, and proceeding until a total of 500 different mRNA identifiable transcripts had been assembled.

### Transcript Assembly and Abundance

Identified reads were used to assemble complete transcripts by the transcriptome walking algorithm (25) using an initial minimum overlap of 80 nucleotides. But increased overlap up to 95 was sometimes required to separate transcripts with common motifs or decreases to 60 overlaps for less abundant transcripts. Walking was always continued to identify the complete protein coding sequence, including both START and STOP codons. The walking steps attempted to identify each nucleotide from overlap of 40 reads and then used the highest quality 20 reads of each 40 for assembly. Single nucleotide polymorphisms were recorded where any alternate nucleotide contributed >10% of the reads, but all the reported transcripts represent the canonical sequences.

Only transcripts with complete reading frames that could be putatively identified by the BLAST algorithm (US National Library of Medicine) were accepted into the final collection of 500. In the process, a total of 79 assembled sequences were separately classified as noncoding, partial, or unknown transcripts.

Relative abundances of transcribed mRNA sequences in the two tissues were estimated by searching both complete groomed transcriptome libraries for reads matching the reading frame of each transcript, using the criterion of at least 90/100 identical nucleotide matches to score each read as derived from that transcript. Matching reads as a fraction of total reads counted were then normalized by reading frame length and expressed as abundance relative to the 40S ribosomal protein SA abundance in each transcriptome. This method has previously been found to agree closely with relative abundances estimated by quantitative PCR (26).

### Fitting Relative Abundance Data

**Figure 2.**
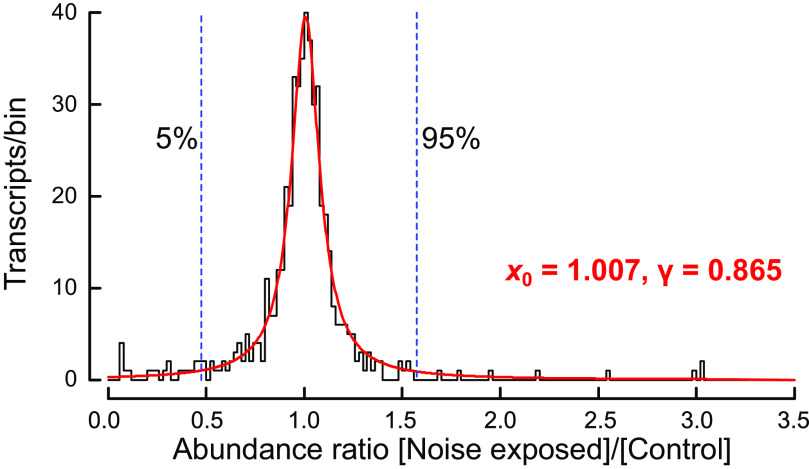
Distribution of ratios of abundances of mRNA transcripts from Müller’s organs of control and noise-exposed locusts. Ratio values were counted into histogram bins of 0.2 width. The continuous line shows the best fitting Cauchy distribution (*Eq. 1*) with parameters *x*_0_ = 1.007, γ = 0.865. Dashed vertical lines indicate the 5% and 95% values of the normalized cumulative distribution (*Eq. 2*) using the same fitted parameters. Values below and above unity correspond to transcripts with reduced and increased abundance in noise-exposed animals, respectively. *x*_0_, location parameter; γ, half width at half maximum.

Abundance ratio values (Noise exposed/Control) were counted into histogram bins of 0.2 width (Fig. 2). The complete histogram was fitted by the Cauchy distribution (27):

(*1*) $$f\left(x\right)\;=A/\left(1+{\left(\left(x-x_{0}\right)/\gamma \right)}^{2}\right)$$

where *x* is abundance ratio, *x*_0_ is the location parameter, γ is the half width at half maximum, and *A* is the value of *f*(*x*) at *x* = *x*_0_. Fitting was performed using a minimum squared error method. Confidence intervals were obtained from the normalized cumulative distribution function, *F*(*x*), at the desired values by successive approximation, using the fitted values of *x*_0_ and γ:

(*2*) $$F\left(x\right)\;=\;0.5\;+\;\left(1/\pi \right)\;tan^{-1}\left(\left(x-x_{0}\right)/\gamma \right)$$

All transcript discovery, assembly, abundance estimation, data processing, and fitting was performed by custom written software using the C++ language and desktop computers.

## RESULTS

A total of 500 mRNA transcripts were assembled to include the complete amino acid reading frame, and in many cases the complete 5′ and 3′ end sequences. Identification codes, abundances in the control and noise-exposed transcriptomes, and putative functions of all transcripts are given in Table 1. Full nucleotide sequences, reading frames, translations, and single nucleotide polymorphisms for all transcripts are available at http://asf-pht.medicine.dal.ca/SCH_Web/. Reading frames ranged from 159 to 15,450 nucleotides (53–5,150 amino acids) with average length 1,927 nucleotides. Based on hypotheses from previous studies (3, 7), we noted that the list of transcripts included seven mechanically activated ion channels, 32 transmembrane transporters or pumps, 24 voltage- or ligand-activated ion channels, and 66 transcription or translation factors.

**Table 1.** Schistocerca gregaria mRNA transcripts and abundances for control and noise-exposed animals

ID Code	GenBank	Control	Noise	Putative Function
SCH_0001	MW962393	−2.432	−2.253	Actin 1
SCH_0002	MK962884	−2.298	−2.272	TRPV cation channel Inactive
SCH_0003	MW962394	−0.374	−0.347	GAPDH
SCH_0004	MW962395	0.583	0.635	Actin 2
SCH_0005	MW962396	−2.804	−2.849	Adenylate cyclase type 2
SCH_0006	MW962397	−1.441	−1.643	Actin 3
SCH_0007	MW962398	−3.451	−3.396	Ankyrin repeat and death containing protein
SCH_0008	MW962399	−2.916	−2.959	TRPN cation channel NompC
SCH_0009	MW962400	−2.739	−2.785	Ankyrin repeat containing 27-like
SCH_0010	MW962401	−1.700	−1.781	Ankyrin repeat containing 54-like
SCH_0011	MW962402	−2.242	−2.259	Mariner Mos1 transpoase
SCH_0012	MW962403	−3.100	−3.161	Distal antennal-like
SCH_0013	MW962404	−1.673	−1.650	Basement membrane-specific heparan sulfate proteoglycan core protein
SCH_0014	MW962405	−1.893	−1.883	NDUFAF4
SCH_0015	MW962406	−0.369	−0.877	Apolipophorin precursor
SCH_0016	MK962885	−2.172	−2.135	TRPV cation channel Nanchung
SCH_0017	MK962886	−2.258	−2.232	Piezo
SCH_0018	MW962407	−1.719	−1.717	Synaptophysin
SCH_0019	MW962408	−1.616	−1.588	Synaptotagmin
SCH_0020	MW962409	−1.169	−1.186	Synaptosomal-Associated Protein SNAP 25
SCH_0021	MW962410	−1.949	−1.981	Transmembrane channel TMC 7
SCH_0022	MW962411	−0.525	−0.487	Na/K ATPase
SCH_0023	MW962412	−2.086	−2.202	Solute carrier family 12
SCH_0024	MW962413	−1.964	−1.857	Bumetanide-sensitive K/Na/Cl transporter
SCH_0025	MW962414	−1.971	−2.015	Bumetanide-sensitive K/Na/Cl transporter
SCH_0026	MW962415	−2.400	−2.543	Na/H Exchanger
SCH_0027	MW962416	−3.193	−3.057	Na/H Exchanger
SCH_0028	MW962417	−1.697	−1.736	Ca-transporting ATPase
SCH_0029	MW962418	−3.319	−3.064	Na/H Exchanger
SCH_0030	MW962419	−2.423	−2.442	K channel Shaker
SCH_0031	MW962420	−1.965	−1.985	G-protein activated IR K channel
SCH_0032	MW962421	0.150	0.243	Tubulin alpha
SCH_0033	MW962422	0.194	0.236	Tubulin alpha
SCH_0034	MW962423	−0.143	−0.127	Tubulin alpha
SCH_0035	MW962424	−1.066	−1.081	Spectrin alpha chain
SCH_0036	MW962425	−2.797	−2.911	EAG K channel
SCH_0037	MW962426	−1.675	−1.654	MAP kinase-activated protein kinase
SCH_0038	MW962427	−1.748	−1.840	CaM kinase II
SCH_0039	MW962428	−1.588	−1.665	Protein kinase DC2
SCH_0040	MW962429	−2.095	−2.168	DENN domain containing protein
SCH_0041	MW962430	−1.714	−1.809	Carboxylesterase
SCH_0042	MW962431	−0.585	−0.472	Carboxylesterase
SCH_0043	MW962432	−0.270	−0.185	Aquaporin
SCH_0044	MW962433	−0.973	−2.136	Carboxylesterase
SCH_0045	MW962434	−1.152	−1.093	Carboxylesterase
SCH_0046	MW962435	−0.330	−0.200	Carboxylesterase
SCH_0047	MW962436	−0.756	−0.680	Carboxylesterase
SCH_0048	MW962437	−1.345	−1.282	Carboxylesterase
SCH_0049	MW962438	−1.279	−1.375	Carboxylesterase
SCH_0050	MW962439	−2.129	−2.077	Carboxylesterase
SCH_0051	MW962440	−1.214	−1.101	Carboxylesterase
SCH_0052	MW962441	−2.081	−2.116	Acetylcholine esterase
SCH_0053	MW962442	−1.773	−1.798	Serine-threonine protein phosphatase II
SCH_0054	MW962443	−1.726	−1.754	Serine-threonine protein phosphatase II
SCH_0055	MW962444	−1.974	−2.065	Beta-arrestin 1
SCH_0056	MW962445	−1.717	−1.623	3-phosphoinositide-dependent protein kinase
SCH_0057	MW962446	−2.408	−2.441	5-AMP-activated protein kinase catalytic subunit α2
SCH_0058	MW962447	−2.200	−2.247	Calcium/calmodulin-responsive adenylate cyclase
SCH_0059	MW962448	−2.440	−2.482	Adenylate cyclase type 9
SCH_0060	MW962449	−2.892	−2.892	Adenylate cyclase type 8
SCH_0061	MW962450	−1.786	−1.794	Ankyrin 3
SCH_0062	MW962451	−2.146	−2.175	Ankyrin 3
SCH_0063	MW962452	−1.272	−1.262	Argonaute 2
SCH_0064	MW962453	−2.462	−2.519	Argonaute 2
SCH_0065	MW962454	−2.484	−2.627	Argonaute 1
SCH_0066	MW962455	−2.708	−2.664	G protein-activated IR K channel
SCH_0067	MW962456	−1.809	−1.901	Spectrin beta chain
SCH_0068	MW962457	−2.955	−2.871	Ca-activated K channel slowpoke
SCH_0069	MW962458	−0.597	−0.586	ATP-dependent RNA helicase
SCH_0070	MW962459	−0.583	−0.584	ATP-dependent RNA helicase
SCH_0071	MW962460	−0.372	−0.425	ATP-dependent RNA helicase
SCH_0072	MW962461	−0.901	−0.911	Basigin
SCH_0073	MW962462	−0.737	−0.859	C-type lysozyme
SCH_0074	MW962463	−0.356	−0.306	Calmodulin
SCH_0075	MW962464	−0.626	−0.606	Calmodulin
SCH_0076	MW962465	−1.237	−1.262	Calpain
SCH_0077	MW962466	−0.435	−0.423	Calreticulin
SCH_0078	MW962467	−2.047	−2.085	Voltage-activated Na channel alpha subunit
SCH_0079	MW962468	−2.117	−2.062	Voltage-activated Cl channel CLC type
SCH_0080	MW962469	−1.551	−1.534	HCN channel
SCH_0081	MW962470	−1.344	−1.407	K channel subfamily K member
SCH_0082	MW962471	−1.622	−1.600	cAMP-dependent protein kinase catalytic subunit
SCH_0083	MW962472	−1.398	−1.438	cAMP-dependent protein kinase type II regulatory subunit
SCH_0084	MW962473	−1.650	−1.611	Cryptochrome 2
SCH_0085	MW962474	−2.302	−2.306	Cryptochrome 2
SCH_0086	MW962475	−1.274	−1.271	CRAC Calcium release-activated calcium channel
SCH_0087	MW962476	−2.151	−2.155	Cyclin-dependent kinase 5
SCH_0088	MW962477	−2.006	−1.982	Diacylglycerol kinase epsilon
SCH_0089	MW962478	−1.788	−1.781	Diacylglycerol kinase theta
SCH_0090	MW962479	−2.007	−1.969	Protein kinase CP
SCH_0091	MW962480	−2.304	−2.345	Dicer 1
SCH_0092	MW962481	−0.204	0.085	Endocuticle structural glycoprotein SgAbd 2
SCH_0093	MW962482	−1.611	−1.661	Epidermal growth factor receptor
SCH_0094	MW962483	−2.856	−2.774	Glycine receptor alpha subunit
SCH_0095	MW962484	−0.706	−0.581	Phe-4-monooxygenase (Henna)
SCH_0096	MW962485	−2.269	−2.281	Huntingtin
SCH_0097	MW962486	−2.111	−2.169	IP3 receptor
SCH_0098	MW962487	−2.465	−2.557	Peripheral plasma membrane protein CASK
SCH_0099	MW962488	−1.929	−1.939	E3 ubiquitin-protein ligase parkin
SCH_0100	MW962489	−1.711	−1.705	Serine/threonine-protein kinase Tricorner
SCH_0101	MW962490	−2.165	−2.172	Serine/threonine-protein kinase Warts
SCH_0102	MW962491	−2.215	−2.301	Ribosomal protein S6 kinase beta
SCH_0103	MW962492	−1.726	−1.672	1-phosphatidylinositol 4,5-bisphosphate phosphodiesterase
SCH_0104	MW962493	−2.449	−2.571	1-phosphatidylinositol 4,5-bisphosphate phosphodiesterase
SCH_0105	MW962494	−1.142	−1.131	Na/H exchange regulatory cofactor
SCH_0106	MZ004840	−0.053	−0.012	NADH dehydrogenase subunit 1 (mitochondrial)
SCH_0107	MW962495	−2.124	−2.149	Phosphatidylinositol 3,4,5-trisphosphate 3- phosphatase
SCH_0108	MW962496	−0.122	−0.087	14-3-3 protein zeta
SCH_0109	MW962497	−0.682	−0.656	14-3-3 protein epsilon
SCH_0110	MW962498	−1.890	−1.963	5-oxoprolinase
SCH_0111	MW962499	−1.308	−1.327	Cation/H exchanger NHE
SCH_0112	MW962500	−1.544	−1.508	V-type proton ATPase subunit H
SCH_0113	MW962501	−1.189	−1.146	V-type proton ATPase subunit B
SCH_0114	MW962502	−1.145	−1.136	V-type proton ATPase catalytic subunit A
SCH_0115	MW962503	−1.152	−1.143	V-type proton ATPase subunit D
SCH_0116	MW962504	−0.864	−0.828	V-type proton ATPase subunit E
SCH_0117	MW962505	−1.747	−1.744	Calcium permeable stress-gated cation channel
SCH_0118	MW962506	−0.376	−0.268	Annexin B9
SCH_0119	MW962507	−0.902	−0.900	Annexin B9
SCH_0120	MW962508	−1.512	−1.494	Annulin
SCH_0121	MW962509	−1.336	−1.458	Hemocyte protein-glutamine γ-glutamyltransferase
SCH_0122	MW962510	−1.718	−1.683	Anoctamin (Ca-activated Cl channel)
SCH_0123	MW962511	−2.312	−2.347	Anoctamin (Ca-activated Cl channel)
SCH_0124	MW962512	−1.196	−1.103	Carbonic anhydrase
SCH_0125	MW962513	−0.865	−1.010	Carbonic anhydrase
SCH_0126	MW962514	−1.681	−1.774	Carbonic anhydrase
SCH_0127	MW962515	−0.234	−0.242	Eukaryotic initiation factor 4 A-II
SCH_0128	MW962516	−1.192	−1.420	Attractin-like
SCH_0129	MW962517	−2.724	−2.957	Attractin-like
SCH_0130	MW962518	−1.304	−1.205	Collagen alpha chain
SCH_0131	MW962519	−1.874	−1.829	Collagen alpha chain
SCH_0132	MW962520	−1.455	−1.373	Collagen apha chain
SCH_0133	MW962521	−1.931	−1.918	Chromatin-remodeling ATPase INO80
SCH_0134	MW962522	−2.197	−2.237	Helicase domino
SCH_0135	MW962523	−2.073	−2.016	Helicase-like
SCH_0136	MW962524	−2.231	−2.111	Eyes absent
SCH_0137	MW962525	−2.114	−2.312	Ecdysone receptor
SCH_0138	MW962526	−1.723	−1.872	Retinoid-X receptor
SCH_0139	MW962527	−2.500	−2.640	Embryonic gonad like
SCH_0140	MW962528	−2.690	−2.667	Nuclear hormone receptor
SCH_0141	MW962529	−2.745	−2.742	Nuclear hormone receptor
SCH_0142	MW962530	−2.109	−2.061	Early growth response protein
SCH_0143	MW962531	−3.147	−3.133	Tyramine receptor
SCH_0144	MW962532	0.141	0.332	Endocuticle structural glycoprotein SgAbd 4
SCH_0145	MW962533	−1.460	−1.232	Endocuticle structural glycoprotein SgAbd 3
SCH_0146	MW962534	−2.996	−3.032	Na/Ca exchanger
SCH_0147	MW962535	−2.210	−2.238	Na/H Exchanger
SCH_0148	MW962536	−2.184	−2.207	GABA A receptor beta subunit
SCH_0149	MW962537	−1.654	−1.560	Glutamate-gated chloride channel
SCH_0150	MW962538	−2.410	−2.447	Glutamate-gated chloride channel
SCH_0151	MW962539	−2.684	−2.754	GABA B receptor subunit 1
SCH_0152	MW962540	−2.554	−2.545	Choline acetyltransferase
SCH_0153	MW962541	−3.304	−3.440	GABA B receptor subunit 2
SCH_0154	MW962542	−2.235	−2.238	Sodium bicarbonate cotransporter
SCH_0155	MW962543	−1.437	−1.524	Band 3 anion transporter
SCH_0156	MW962544	−1.118	−1.194	Dystonin
SCH_0157	MW962545	−1.277	−1.265	Microtubule-associated protein
SCH_0158	MW962546	−1.387	−1.379	Microtubule-associated serine-threonine kinase
SCH_0159	MW962547	−2.178	−2.221	Microtubule-associated serine-threonine kinase
SCH_0160	MW962548	−2.142	−2.154	Serine-threonine kinase sgk-like
SCH_0161	MW962549	−1.573	−1.527	Serine-threonine kinase grp
SCH_0162	MW962550	−1.913	−1.881	Tubulin gamma
SCH_0163	MW962551	−1.890	−2.230	Myosin heavy chain
SCH_0164	MW962552	−1.899	−2.155	Myosin light chain
SCH_0165	MW962553	−1.991	−2.015	TRPML3 (mucolipin 3)
SCH_0166	MW962554	−3.189	−3.174	Pickpocket (ENaC, ASIC family)
SCH_0167	MW962555	−2.869	−2.891	Glutamate-gated chloride channel
SCH_0168	MW962556	−2.171	−2.285	Transcriptional repressor Scratch
SCH_0169	MW962557	−2.176	−2.182	Zinc finger protein 432-like
SCH_0170	MW962558	−2.308	−2.339	Locust corazonin-related transcriptional factor
SCH_0171	MW962559	−1.963	−1.960	Zinc finger protein 271-like
SCH_0172	MW962560	−1.629	−1.602	Zinc finger protein 271-like
SCH_0173	MW962561	−2.338	−2.367	Zinc finger protein 236-like
SCH_0174	MW962562	−2.115	−2.107	Zinc finger protein 62-like
SCH_0175	MW962563	−2.560	−2.489	Zinc finger protein 341-like
SCH_0176	MW962564	−2.368	−2.308	Zinc finger protein 271-like
SCH_0177	MW962565	−2.430	−2.368	Zinc finger protein 813-like
SCH_0178	MW962566	−2.251	−2.220	Zinc finger protein 32-like
SCH_0179	MW962567	−2.218	−2.218	Zinc finger protein 135-like
SCH_0180	MW962568	−2.333	−2.358	Zinc finger protein 569-like
SCH_0181	MW962569	−1.986	−2.012	Zinc finger protein 2-like
SCH_0182	MW962570	−1.899	−1.917	Dicer 2
SCH_0183	MW962571	−1.449	−1.455	Eukaryotic initiation factor 3 A
SCH_0184	MW962572	0.378	0.378	Elongation factor 1 alpha
SCH_0185	MW962573	−1.107	−1.079	Eukaryotic initiation factor 2 subunit 1
SCH_0186	MW962574	−2.109	−2.095	DSCAM 2
SCH_0187	MW962575	−2.137	−2.150	Dynamin
SCH_0188	MW962576	−2.143	−2.154	Dynamin
SCH_0189	MW962577	−1.793	−1.791	Dynamin
SCH_0190	MW962578	−2.027	−2.053	Enhancer of sevenless 2B
SCH_0191	MW962579	−1.190	−1.188	Lamin Dm0
SCH_0192	MW962580	−1.867	−1.853	E3 ubuquitin-protein kinase RNF123
SCH_0193	MW962581	−0.711	−0.670	Voltage-dependent anion channel
SCH_0194	MW962582	−1.677	−1.673	L-type calcium channel beta subunit
SCH_0195	MW962583	−1.863	−1.868	L-type calcium channel beta subunit
SCH_0196	MW962584	−0.654	−0.602	Chitin deacetylase
SCH_0197	MW962585	−0.490	−0.349	Chitin deacetylase
SCH_0198	MW962586	−1.079	−0.987	Chitin deacetylase
SCH_0199	MW962587	−1.911	−2.319	Troponin
SCH_0200	MW962588	−1.456	−1.505	Alpha actinin
SCH_0201	MW962589	−1.197	−1.200	Spectrin beta chain
SCH_0202	MW962590	−1.471	−1.460	Lola - longitdinals lacking
SCH_0203	MW962591	−1.459	−1.483	Bric-a-brac-like
SCH_0204	MW962592	−1.147	−1.162	BTG 2
SCH_0205	MW962593	−1.572	−1.593	BTG 3
SCH_0206	MW962594	−1.518	−1.547	Calcium-transporting ATPase
SCH_0207	MW962595	−2.647	−2.688	Neural cadherin
SCH_0208	MW962596	0.719	−0.424	Vitellogenin A
SCH_0209	MW962597	−0.872	−0.875	Ubiquitin-conjugating enzyme E2-17 kDA
SCH_0210	MZ004841	0.864	0.877	Cytochrome B (mitochondrial)
SCH_0211	MW962598	−0.836	−0.816	Ras-related protein Rab 1 A
SCH_0212	MZ004842	1.323	1.341	Cytochrome c oxidase subunit 1 (mitochondrial)
SCH_0213	MW962599	0.352	0.358	ATP-ADP translocator
SCH_0214	MW962600	−0.304	−0.229	Arginine kinase
SCH_0215	MW962601	0.622	0.194	Hexamerin-like
SCH_0216	MW962602	−0.150	−0.129	40S ribosomal protein S4
SCH_0217	MW962603	−0.166	−0.198	Polyubiquitin
SCH_0218	MW962604	−0.457	−0.357	Heat shock protein 90
SCH_0219	MW962605	0.235	0.062	Imaginal disc growth factor
SCH_0220	MW962606	0.348	0.355	Tubulin beta
SCH_0221	MW962607	−0.478	−0.535	Superoxide dismutase [Cu-Zn]
SCH_0222	MW962608	−1.530	−1.607	Lacunin
SCH_0223	MZ004843	0.999	1.009	ATP synthase F0 subunit 6 (mitochondrial)
SCH_0224	MZ004844	0.919	0.941	Cytochrome c oxidase subunit 2 (mitochondrial)
SCH_0225	MW962609	0.376	0.360	Icarapin-like
SCH_0226	MZ004845	0.976	1.002	Cytochrome c oxidase subunit 3 (mitochondrial)
SCH_0227	MW962610	0.687	−0.458	Vitellogenin B
SCH_0228	MW962611	0.437	−0.580	Hexamerin-like
SCH_0229	MW962612	0.000	0.000	40S ribosomal protein SA
SCH_0230	MW962613	−0.979	−0.972	Sortilin-related receptor
SCH_0231	MW962614	−0.782	−0.597	Pacifastin-related peptide precursor
SCH_0232	MW962615	−0.120	−0.019	Transferrin
SCH_0233	MW962616	0.317	−0.216	Hexamerin-like
SCH_0234	MW962617	−0.348	−0.268	Thioredoxin 2-like
SCH_0235	MW962618	0.514	0.557	Heat shock protein 70
SCH_0236	MW962619	−0.394	−0.427	Cytochrome c oxidase subunit 5
SCH_0237	MW962620	−0.206	−0.181	60S ribosomal protein L19
SCH_0238	MW962621	−1.711	−1.663	Solute carrier family 25 member 44
SCH_0239	MW962622	−0.346	−0.333	ATP synthase subunit alpha (mitochondrial)
SCH_0240	MW962623	−0.774	−0.723	NDRG3
SCH_0241	MW962624	0.100	0.123	Activating transcription factor of chaperone
SCH_0242	MW962625	−0.606	−0.618	Ly 6 neurotoxin
SCH_0243	MW962626	−2.951	−3.012	Glutamate receptor, ionotropic
SCH_0244	MW962627	0.081	0.039	Ferritin heavy subunit
SCH_0245	MW962628	−0.720	−0.969	Facilitated trehalose transporter
SCH_0246	MW962629	−0.910	−0.834	Innexin 2
SCH_0247	MW962630	−0.087	−0.440	Gamma butyrobetaine dioxygenase
SCH_0248	MW962631	−0.376	−0.355	Ly 6 neurotoxin
SCH_0249	MW962632	0.045	0.137	Tubulin beta
SCH_0250	MW962633	−0.215	−0.162	Midline fasciclin
SCH_0251	MW962634	−1.321	−1.316	GTP-binding protein sar1
SCH_0252	MW962635	−0.492	−0.544	Legumain
SCH_0253	MW962636	−1.576	−1.597	Membrane-associated protein sar1
SCH_0254	MW962637	−1.933	−1.906	Exocyst complex component 5
SCH_0255	MW962638	−1.983	−2.577	GILT-like
SCH_0256	MW962639	−1.744	−1.850	Fibrillin 2
SCH_0257	MW962640	−1.395	−1.402	CLCN3 H/Cl exchange transporter 3
SCH_0258	MW962641	−1.209	−1.182	DNA topoisomerase (mitochondrial)
SCH_0259	MW962642	−1.448	−1.420	Farnesol dehydrogenase
SCH_0260	MW962643	−1.704	−1.750	CLUH Clustered mitochondrial protein homolog
SCH_0261	MW962644	−1.292	−1.282	Alpha-2-macroglobulin receptor-associated protein
SCH_0262	MW962645	−0.618	−0.638	Integral membrane protein 2 C
SCH_0263	MW962646	−1.654	−1.651	Isocitrate dehydrogenase [NAD] γ-subunit (mitochondrial)
SCH_0264	MW962647	−0.967	−0.959	Phosphoglycerate mutase 2
SCH_0265	MW962648	0.215	0.214	Translationally-controlled tumor protein
SCH_0266	MW962649	−0.651	−0.678	ADP-ribosylation factor 1
SCH_0267	MW962650	−1.525	−1.548	Solute carrier family 22
SCH_0268	MW962651	−0.026	0.039	Defense protein
SCH_0269	MW962652	−1.389	−1.407	Integrin
SCH_0270	MW962653	−0.951	−1.005	Nose resistant to fluoxetine protein 6
SCH_0271	MW962654	−1.514	−1.491	GTP-binding protein 128up
SCH_0272	MW962655	−0.921	−0.912	Succinate dehydrogenase flavoprotein subunit (mitochondrial)
SCH_0273	MW962656	−0.320	−0.196	Spermine synthase
SCH_0274	MW962657	−1.188	−1.163	Carbohydrate sulfotransferase 11
SCH_0275	MW962658	−2.080	−2.060	Uridine-cytidine kinase-like 1
SCH_0276	MW962659	−0.435	−0.437	Protein krasavietz
SCH_0277	MW962660	−0.114	−0.394	Nuclear protein 1
SCH_0278	MW962661	0.074	0.085	Elongation factor 2
SCH_0279	MW962662	−0.610	−0.765	Peroxiredoxin 6
SCH_0280	MW962663	0.039	0.074	Chemosensory protein precursor
SCH_0281	MW962664	−0.063	−0.102	Polyadenylate-binding protein 1
SCH_0282	MW962665	−0.712	−0.699	Poly(U)-specific endoribonuclease
SCH_0283	MW962666	−1.029	−0.957	Sodium-dependent phosphate transporter 1-A
SCH_0284	MW962667	−1.285	−1.252	*N*-acetyltransferase san
SCH_0285	MW962668	−1.266	−1.287	ABC transporter G family member 23
SCH_0286	MW962669	−1.983	−1.994	Tetratricopeptide Repeat TANC2
SCH_0287	MW962670	−1.538	−1.602	Transmembrane protein 53
SCH_0288	MW962671	−1.027	−1.397	Pancreatic lipase-related protein 2
SCH_0289	MW962672	−1.188	−1.204	Singed
SCH_0290	MW962673	0.212	−0.104	Apolipophorin III
SCH_0291	MW962674	−1.744	−1.810	Myelin regulatory factor
SCH_0292	MW962675	−0.862	−0.841	CHCHD10 (mitochondrial)
SCH_0293	MW962676	−0.574	−0.502	Dynein light chain A
SCH_0294	MW962677	−1.476	−1.491	Transcription factor CP2
SCH_0295	MW962678	−0.435	−0.438	Profilin
SCH_0296	MW962679	−1.549	−1.537	Nicastrin
SCH_0297	MW962680	−0.390	−0.415	Inhibitor of apoptosis
SCH_0298	MW962681	−0.612	−0.573	Leupaxin
SCH_0299	MW962682	−0.687	−0.669	Transcription factor BTF3
SCH_0300	MW962683	−1.268	−1.325	Atlastin
SCH_0301	MW962684	−0.873	−0.877	Beta-N-acetylglucosaminidase
SCH_0302	MW962685	−0.311	−0.219	Endocuticle structural glycoprotein SgAbd 5
SCH_0303	MW962686	−0.030	−0.073	Ferritin subunit
SCH_0304	MW962687	−1.159	−1.139	Actin-binding LIM protein 3
SCH_0305	MW962688	−0.240	−0.288	Fructose 1,6-bisphosphate aldolase
SCH_0306	MZ004846	−0.114	−0.283	NADH dehydrogenase subunit 6 (mitochondrial)
SCH_0307	MW962689	−0.289	−0.255	40S ribosomal protein S24
SCH_0308	MW962690	−0.232	−0.057	Chemosensory protein CSP-sg4
SCH_0309	MW962691	−0.850	−0.864	Double-stranded RNA-binding protein Staufen
SCH_0310	MW962692	−0.246	−0.197	Myophilin
SCH_0311	MW962693	−0.246	−0.242	ATP-synthase subunit beta
SCH_0312	MW962694	−2.208	−2.197	Aminopeptidase N
SCH_0313	MW962695	−1.770	−1.750	Tyrosine-protein phosphatase non-receptor type 1
SCH_0314	MW962696	−1.938	−1.892	Tyrosine-protein phosphatase non-receptor type 9
SCH_0315	MW962697	−1.831	−1.853	Tyrosine-protein phosphatase Lar
SCH_0316	MW962698	−2.161	−2.187	Tyrosine-protein phosphatase non-receptor type 4
SCH_0317	MW962699	−1.932	−1.938	Tyrosine-protein phosphatase non-receptor type 69 D
SCH_0318	MW962700	−1.871	−1.847	Tyrosine-protein phosphatase non-receptor type 99 A
SCH_0319	MW962701	−1.970	−2.238	Tyrosine-protein phosphatase non-receptor type 5-like
SCH_0320	MW962702	−1.762	−1.729	Receptor-type tyrosine-protein phosphatase N2
SCH_0321	MW962703	−2.517	−2.642	Tyrosine−protein phosphatase non-receptor type 14
SCH_0322	MW962704	−1.642	−1.652	Presenilin
SCH_0323	MW962705	−1.443	−1.444	Zinc finger protein 330 homolog
SCH_0324	MW962706	−0.788	−0.306	Chemosensory protein precursor
SCH_0325	MW962707	−0.240	−0.224	60S ribosomal protein L36
SCH_0326	MW962708	−0.400	−0.366	60S ribosomal protein L18a
SCH_0327	MW962709	−0.879	−0.739	Serpin
SCH_0328	MW962710	−0.733	−0.764	Eukaryotic translation initiation factor 4 gamma 2
SCH_0329	MW962711	−1.852	−1.812	Mitochondrial intermediate peptidase
SCH_0330	MW962712	−1.207	−1.176	Eukaryotic translation initiation factor 3 subunit D
SCH_0331	MW962713	−1.397	−1.616	Cystathionine beta-synthase
SCH_0332	MW962714	−0.140	−0.796	Hexamerin-like
SCH_0333	MW962715	−1.296	−1.313	rap1 GTPase-activating protein 1
SCH_0334	MW962716	−2.852	−2.910	Glutamate receptor, ionotropic
SCH_0335	MW962717	−2.721	−2.817	Nicotinic acetylcholine receptor, beta subunit
SCH_0336	MW962718	−2.336	−2.349	Nicotinic acetylcholine receptor, alpha subunit
SCH_0337	MW962719	−1.228	−1.301	Aldehyde dehydrogenase
SCH_0338	MW962720	−1.435	−1.439	Coatomer subunit delta
SCH_0339	MW962721	−0.576	−0.543	Histone H3
SCH_0340	MW962722	−1.256	−1.226	Proton-coupled amino acid transporter-like
SCH_0341	MW962723	−0.912	−0.921	Ras-related protein rab7
SCH_0342	MW962724	−1.463	−1.462	Programmed cell death protein 10
SCH_0343	MW962725	−1.666	−1.639	Adenosylhomocysteinase
SCH_0344	MW962726	−0.992	−1.033	ATP-citrate synthase
SCH_0345	MW962727	−1.307	−1.279	Armadillo
SCH_0346	MW962728	−1.591	−1.579	Enoyl-[acyl-carrier-protein] reductase
SCH_0347	MW962729	−0.936	−1.049	Vigilin
SCH_0348	MW962730	−0.487	−0.495	Peptidyl-prolyl cis-trans isomerase
SCH_0349	MZ004847	0.020	0.050	NADH dehydrogenase subunit 2 (mitochondrial)
SCH_0350	MW962731	−0.796	−0.316	Chemosensory protein
SCH_0351	MW962732	−0.639	−0.661	DnaJ subfamily A member 2
SCH_0352	MW962733	−1.034	−1.052	Angiotensin-converting enzyme
SCH_0353	MW962734	−0.760	−0.735	Myosin regulatory light chain sqh
SCH_0354	MW962735	−0.920	−0.855	Succinate dehydrogenase
SCH_0355	MW962736	−1.413	−1.372	Glucose-induced degradation protein 8
SCH_0356	MW962737	−0.965	−0.984	Nascent polypeptide-associated complex α-subunit
SCH_0357	MW962738	−0.898	−0.899	Splicing factor U2AF 50 kDa subunit
SCH_0358	MW962739	−1.581	−1.445	Proton-coupled amino acid transporter-like CG1139
SCH_0359	MW962740	−0.240	−0.212	40S ribosomal protein S10-like
SCH_0360	MW962741	−0.341	−0.349	Prosaposin
SCH_0361	MW962742	−0.652	−0.709	Cathepsin B
SCH_0362	MW962743	−0.201	−0.865	Hexamerin-like
SCH_0363	MW962744	−1.349	−1.514	Cysteine sulfinic acid decarboxylase
SCH_0364	MW962745	−1.276	−1.278	Peroxisomal biogenesis factor 19
SCH_0365	MW962746	−0.876	−0.845	Heterogeneous nuclear ribonucleoprotein K
SCH_0366	MW962747	−2.299	−2.376	Inositol polyphosphate 5-phosphatase K
SCH_0367	MW962748	−0.410	−0.455	Ornithine decarboxylase
SCH_0368	MW962749	−0.168	−0.123	60S ribosomal protein L7a
SCH_0369	MW962750	−1.603	−1.589	ATP-dependent RNA helicase DDX54
SCH_0370	MW962751	−1.714	−1.708	Apoptosis-inducing factor 1, mitochondrial
SCH_0371	MW962752	−0.943	−0.899	DNA-directed RNA polymerases I, II, and III subunit RPABC3
SCH_0372	MW962753	−0.306	−0.294	ADP, ATP carrier protein
SCH_0373	MW962754	−1.065	−1.082	ATP-dependent RNA helicase dbp2
SCH_0374	MW962755	−1.408	−1.473	Aspartate aminotransferase, cytoplasmic-like
SCH_0375	MW962756	−1.550	−1.503	Protein 5NUC
SCH_0376	MW962757	−0.702	−0.604	Sigma glutathione S-transferase
SCH_0377	MW962758	−1.787	−1.745	DEAD-box helicase Dbp80
SCH_0378	MW962759	−1.644	−1.642	Hydroxyacylglutathione hydrolase, mitochondrial
SCH_0379	MW962760	−2.156	−2.098	Ribitol-5-phosphate xylosyltransferase 1
SCH_0380	MW962761	−1.381	−1.359	Dihydropyrimidinase
SCH_0381	MW962762	−0.716	−0.648	Protein stunted
SCH_0382	MW962763	−0.799	−0.737	Cytochrome b reductase 1
SCH_0383	MW962764	−1.407	−1.324	Eukaryotic translation initiation factor 3 subunit K
SCH_0384	MW962765	−1.562	−1.567	WASH complex subunit 3
SCH_0385	MW962766	−0.319	−0.302	40S ribosomal protein S16
SCH_0386	MW962767	−0.356	−0.329	60S ribosomal protein L15
SCH_0387	MW962768	−1.392	−0.916	Lysozyme-like
SCH_0388	MW962769	−1.403	−1.376	Proteasome subunit alpha type-2
SCH_0389	MW962770	−0.607	−0.556	Protein tyrosine phosphatase type IVA 1
SCH_0390	MW962771	−1.148	−1.140	Charged multivesicular body protein 4c
SCH_0391	MW962772	−0.516	−0.518	Malate dehydrogenase
SCH_0392	MW962773	−0.953	−0.950	Mid1-interacting protein 1
SCH_0393	MW962774	−0.152	−0.127	60S ribosomal protein L18
SCH_0394	MW962775	−1.344	−1.367	GDAP2 homolog
SCH_0395	MW962776	−0.501	−0.456	Heat shock protein 20.6
SCH_0396	MW962777	−1.294	−1.291	ATP-binding cassette sub-family F member 2
SCH_0397	MW962778	−0.572	−1.071	Prostatic acid phosphatase
SCH_0398	MW962779	−0.641	−0.620	S-phase kinase-associated protein 1
SCH_0399	MW962780	−0.242	−0.210	60S ribosomal protein L8
SCH_0400	MW962781	−1.362	−1.388	Actin-related protein 2/3 complex subunit 5-like
SCH_0401	MW962782	−1.100	−1.167	Tudor-SN
SCH_0402	MW962783	−1.050	−1.068	CYP450
SCH_0403	MW962784	−1.246	−1.431	Trehalase
SCH_0404	MW962785	−1.132	−1.131	Peroxisomal membrane protein 2
SCH_0405	MW962786	−0.943	−0.954	Angiotensin-converting enzyme
SCH_0406	MW962787	−0.321	−0.298	60S ribosomal protein L4
SCH_0407	MW962788	−0.975	−0.970	Na/K ATPase beta
SCH_0408	MW962789	−0.607	−0.631	Glutathione S-transferase delta
SCH_0409	MW962790	−0.497	−0.472	Phosphate carrier 2
SCH_0410	MW962791	−2.200	−2.239	Phosphate carrier 1
SCH_0411	MW962792	−1.636	−1.625	Transcription factor MafK
SCH_0412	MW962793	−1.334	−1.370	Segmentation protein cap'n'collar
SCH_0413	MW962794	−1.110	−1.057	Ubiquitin-conjugating enzyme E2 i
SCH_0414	MW962795	−1.803	−1.811	Ubiquitin carboxyl-terminal hydrolase 15-like
SCH_0415	MW962796	−1.292	−1.316	Acyl-CoA-binding protein homolog
SCH_0416	MW962797	−1.576	−1.606	Pyroglutamyl-peptidase 1
SCH_0417	MW962798	−1.615	−1.627	Zeta glutathione S-transferase
SCH_0418	MW962799	−2.145	−2.233	SAX-3
SCH_0419	MW962800	−1.540	−1.619	5-aminolevulinate synthase, erythroid-specific, mitochondrial
SCH_0420	MW962801	−0.923	−0.922	Nucleobindin-2
SCH_0421	MW962802	−1.989	−2.039	Dyslexia-associated protein KIAA0319-like
SCH_0422	MW962803	−0.604	−0.601	Peptidyl-prolyl cis-trans isomerase 5
SCH_0423	MW962804	−0.513	−0.489	Thymosin
SCH_0424	MW962805	−1.308	−1.256	Fatty acyl-CoA reductase
SCH_0425	MW962806	−1.289	−1.231	T-complex protein subunit eta
SCH_0426	MW962807	−0.609	−1.585	Timeless
SCH_0427	MW962808	−1.071	−1.066	Cuticlin-1
SCH_0428	MW962809	−1.146	−1.140	Eukaryotic translation initiation factor 3 subunit C
SCH_0429	MW962810	−1.330	−1.399	Ig-like and fibronectin type-III domain-containing protein 1
SCH_0430	MW962811	−1.320	−1.314	CD109 antigen-like
SCH_0431	MW962812	−1.545	−1.541	Poly(rC)-binding protein 3
SCH_0432	MW962813	−0.541	−0.555	tRNA (uracil-5-)-methyltransferase
SCH_0433	MW962814	−0.795	−0.804	Enolase
SCH_0434	MW962815	−1.397	−1.401	NAD-dependent protein deacetylase sirtuin-2
SCH_0435	MW962816	−1.603	−1.600	TM2 domain-containing protein CG11103
SCH_0436	MW962817	−1.525	−1.527	Dynein beta chain, ciliary
SCH_0437	MW962818	−0.981	−0.968	Peroxidase
SCH_0438	MW962819	−1.491	−1.487	Phospholipid phosphatase 2
SCH_0439	MW962820	−1.544	−1.586	Draper
SCH_0440	MW962821	−0.378	−0.398	Eukaryotic translation initiation factor 5 A
SCH_0441	MW962822	−0.061	−0.081	40S ribosomal protein S20
SCH_0442	MW962823	−0.286	−0.163	Chemosensory protein
SCH_0443	MW962824	−1.139	−0.964	Cytochrome P450 CYP4G102
SCH_0444	MW962825	−0.908	−0.920	Leucine-rich repeat protein SHOC-2
SCH_0445	MW962826	−0.610	−0.664	Y-box factor homolog
SCH_0446	MW962827	−1.889	−1.857	Zinc transporter 9
SCH_0447	MW962828	−1.604	−1.265	Endocuticle structural glycoprotein SgAbd 2
SCH_0448	MW962829	−1.744	−1.338	Endocuticle structural glycoprotein SgAbd 8
SCH_0449	MW962830	−1.290	−1.286	Eukaryotic translation initiation factor 3 subunit B
SCH_0450	MW962831	−1.217	−1.153	Apyrase
SCH_0451	MW962832	−1.550	−1.539	Myotubularin-related protein 9
SCH_0452	MW962833	−1.067	−1.077	Ras-related protein Rab-5C
SCH_0453	MW962834	−1.482	−1.453	Proteasome subunit alpha type-6
SCH_0454	MW962835	−0.649	−0.827	Serine protease 42
SCH_0455	MW962836	−1.732	−1.749	E3 ubiquitin-protein ligase HECTD1
SCH_0456	MW962837	−2.694	−2.668	EHMT2 histone-lysine N-methyltransferase
SCH_0457	MW962838	−0.928	−0.971	Enoyl-CoA hydratase, mitochondrial
SCH_0458	MW962839	−1.312	−1.237	Transcription factor Kayak
SCH_0459	MW962840	−2.248	−2.277	Zinc finger protein 674-like
SCH_0460	MW962841	−3.194	−3.182	DNA methyltransferase 1
SCH_0461	MW962842	−1.041	−1.088	Eukaryotic translation initiation factor 3 subunit J
SCH_0462	MW962843	−0.930	−0.907	Myeloid leukemia factor
SCH_0463	MW962844	−0.717	−0.648	Stathmin
SCH_0464	MW962845	−1.705	−1.741	Gualynate kinase
SCH_0465	MW962846	−1.234	−1.237	Kinase D-interacting substrate of 220 kDA
SCH_0466	MW962847	−1.551	−1.603	Thaumatin-like
SCH_0467	MW962848	−1.010	−1.389	Clavesin-1
SCH_0468	MW962849	−1.486	−1.455	Echinoderm microtubule-associated protein-like
SCH_0469	MW962850	−0.759	−0.774	ras-like GTP-binding protein Rho1
SCH_0470	MW962851	−1.723	−1.743	Structural maintenance of chromosomes protein 4
SCH_0471	MW962852	−1.792	−1.777	Lethal(2) giant larvae protein
SCH_0472	MW962853	−0.530	−0.528	Iron-sulfur cluster assembly scaffold protein IscU
SCH_0473	MW962854	−0.840	−0.799	GABA receptor-associated protein
SCH_0474	MW962855	−0.635	−1.787	Greglin
SCH_0475	MW962856	−1.938	−2.109	Calcium/calmodulin-dependent protein kinase
SCH_0476	MW962857	−1.221	−1.412	D-arabinitol dehydrogenase 1
SCH_0477	MW962858	−0.724	−1.045	Lipoyltransferase 1
SCH_0478	MW962859	−1.115	−1.429	Multifunctional protein ADE2
SCH_0479	MW962860	−1.054	−1.088	Gelsolin
SCH_0480	MW962861	−1.094	−1.086	Upregulated during skeletal muscle growth 5
SCH_0481	MW962862	−0.356	−0.328	Cysteine-rich protein 1
SCH_0482	MW962863	−0.249	−0.210	40S ribosomal protein S7
SCH_0483	MW962864	−0.147	−0.129	40S ribosomal protein S8
SCH_0484	MW962865	−0.180	−0.144	40S ribosomal protein S3a
SCH_0485	MW962866	−1.089	−1.042	Protein D2
SCH_0486	MW962867	−0.500	−0.523	X-box-binding protein 1
SCH_0487	MW962868	−1.197	−1.014	Lipopolysaccharide-induced TNF α-factor
SCH_0488	MW962869	−2.720	−2.827	Dimmed
SCH_0489	MW962870	−3.474	−3.463	Homeobox protein six1
SCH_0490	MW962871	−1.466	−1.467	Dynein heavy chain, cytoplasmic
SCH_0491	MW962872	−0.974	−0.949	Growth hormone-inducible transmembrane protein-like
SCH_0492	MW962873	−0.407	−0.553	MAP kinase-interacting serine/threonine-proteinkinase 1
SCH_0493	MW962874	−0.510	−0.695	Catalase
SCH_0494	MW962875	−0.723	−0.804	Transketolase
SCH_0495	MW962876	−0.836	−0.793	Proteoglycan carrier of wingless
SCH_0496	MW962877	−1.300	−1.362	Lachesin
SCH_0497	MW962878	−0.785	−1.110	Acyl-CoA Delta(11) desaturase
SCH_0498	MW962879	−1.058	−0.993	Obstructor D2
SCH_0499	MW962880	−0.650	−0.654	Merlin (moesin-ezrin-radixin-like)
SCH_0500	MW962881	−1.288	−1.238	Huntingtin-interacting protein K

Abundance values are given as log_10_([mRNA of X]/[mRNA of 40S Ribosomal protein SA]). Functions are taken from the most similar BLAST search using default parameters. A list in alphabetical function order is available at http://asf-pht.medicine.dal.ca/SCH_Web/.

### Distribution of Abundance Ratios

Abundance measurements were obtained by counting all the reads in each transcriptome that had overlapping agreement with a minimum or 90 contiguous nucleotides of the reading frame. The raw average ratio of all abundances (noise exposed/control) was 1.065, indicating close similarity between the general properties of the two transcriptomes. All abundances were normalized by the abundances of 40S Ribosomal SA transcripts in the two transcriptomes, yielding an average normalized ratio of 0.993.

The distribution of abundance ratios was wide, ranging from 0.069 to 3.038 (noise exposed/control), with a narrow peak near 1.0 (Fig. 2). This experimental distribution failed several tests for normality. For example, the Kolmogorov–Smirnov test rejected the null hypothesis for normality with *P* < 0.001, and the Q-Q plot against the normal distribution was strongly nonlinear. In contrast, the Cauchy distribution (*Eq. 1*), which has previously been used for ratios of normally distributed variables (27), gave a close approximation over the entire range with parameters: *x*_0_ = 1.007, and γ = 0.865. The Cauchy distribution has no meaningful mean or variance values, but the median and mode are both equal to *x*_0_.

Given the single transcriptome data from each condition, and the nature of the ratio distribution, it was impossible to assign statistical significance to individual transcript ratios. Instead, we arbitrarily selected extreme low and high ratios in the cumulative distribution function (*Eq. 2*) from the lowest and highest 5% of the fitted distribution. All other ratios were not considered to be different from the expected distribution around 1.0.

### Transcripts Affected by Noise Exposure

**Figure 3.**
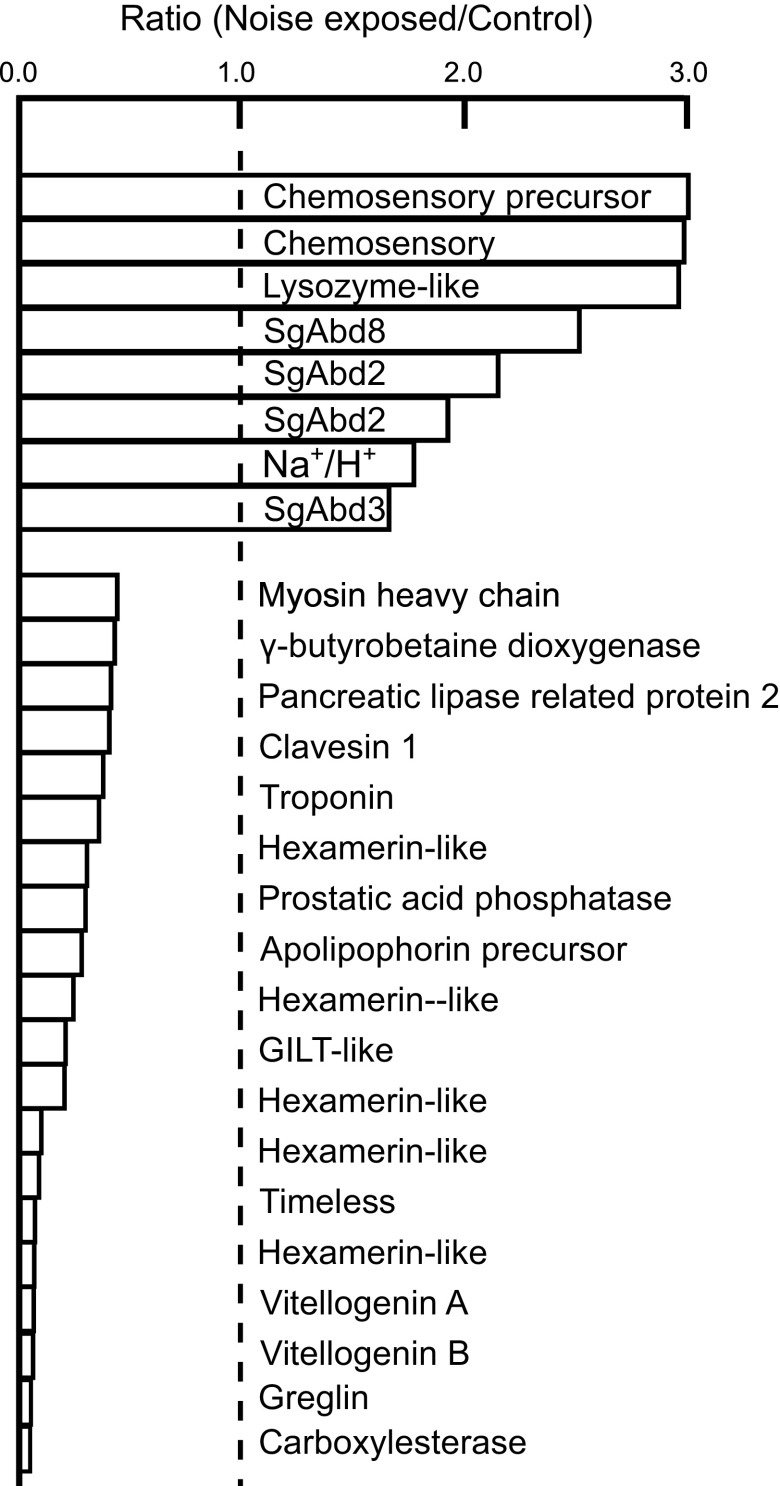
Transcripts at the two tails of the abundance distribution. Numerical values of the abundance ratios are given in Tables 2 and 3. Upper group have ratios above 95% of the cumulative distribution, and lower group have ratios below 5%. Dashed line shows the expected ratio of 1.0 for a transcript unaffected by noise exposure. SgAbd genes are members of the cuticular structural glycoprotein family. Na^+^/H^+^ indicates a sodium/proton ion exchanger. Transcripts with identical gene names have different nucleotide sequences and amino acid sequences (no overlapping reads) but matched genes with the same putative identity by BLAST search.

Transcripts with abundance ratios outside the 5% limits of both tails of the distribution are shown in Fig. 3. Table 2 lists the numerical values of the eight transcripts that we identified as increased by noise exposure. The list includes four endocuticle structural glycoprotein genes, and we note that another member of this gene group (SgAbd4, code: SCH_0144) fell just below the 5% list. The two most elevated transcripts encode a chemosensory protein precursor and a chemosensory protein. Completing the list are one of the four Na^+^/H^+^ exchangers that we found, and a lysozyme-like transcript.

**Table 2.** Schistocerca gregaria mRNA transcripts having increased abundance in noise-exposed animals (abundance ratios >95% of all transcripts)

ID Code	Noise/Control	Putative Function
SCH_0145	1.689	Endocuticle structural glycoprotein SgAbd 3
SCH_0029	1.799	Na/H Exchanger
SCH_0092	1.947	Endocuticle structural glycoprotein SgAbd 2
SCH_0447	2.180	Endocuticle structural glycoprotein SgAbd 2
SCH_0448	2.546	Endocuticle structural glycoprotein SgAbd 8
SCH_0387	2.996	Lysozyme-like
SCH_0350	3.020	Chemosensory protein
SCH_0324	3.038	Chemosensory protein precursor

Table 3 lists the 18 transcripts that were most reduced by noise exposure. The list contains several genes associated with lipid storage and transport (vitellogenins, apolipophorin, gamma butyrobetaine dioxygenase, and pancreatic lipase-related protein). Also reduced were transcripts for protein storage (hexamerins), muscle (troponin and myosin), neuron-related proteins (clavesin and timeless), plus several enzymes with a range of possible functions (carboxylesterase, greglin, GILT-like, and prostatic acid phosphatase).

**Table 3.** Schistocerca gregaria mRNA transcripts having reduced abundance in noise-exposed animals (abundance ratios <95% of all transcripts)

ID Code	Noise/Control	Putative Function
SCH_0044	0.069	Carboxylesterase
SCH_0474	0.071	Greglin
SCH_0227	0.072	Vitellogenin B
SCH_0208	0.072	Vitellogenin A
SCH_0228	0.096	Hexamerin-like
SCH_0426	0.106	Timeless
SCH_0362	0.217	Hexamerin-like
SCH_0332	0.221	Hexamerin-like
SCH_0255	0.255	GILT-like
SCH_0233	0.293	Hexamerin-like
SCH_0015	0.310	Apolipophorin precursor
SCH_0397	0.317	Prostatic acid phosphatase
SCH_0215	0.373	Hexamerin-like
SCH_0199	0.390	Troponin
SCH_0467	0.418	Clavesin-1
SCH_0288	0.427	Pancreatic lipase-related protein 2
SCH_0247	0.444	Gamma butyrobetaine dioxygenase
SCH_0163	0.457	Myosin heavy chain

### Transcription Factors Related to Sound Sensation

The amino acid sequences of the complete list of 66 possible transcription factors were compared by BLAST against the four genes recently associated with sound transduction in a study of age-related *Drosophila* deafness (7). No direct orthologs were identified but three *Drosophila* genes, optix, worniu, and amos, had amino acid sequences with more than 55% identity to locust transcripts (Table 4).

**Table 4.** Drosophila genes linked to age related deafness (7) with the most similar transcripts from Table 1

Drosophila Name	GenBank Code	Locust ID, Name	Identical/Similar
Optix	NP_524695.2	SCH_0489, Six1	57% / 74%
Worniu	NP_476601.1	SCH_0168, Scratch	58% / 69%
Amos	ALC39557.1	SCH_0488, Dimmed	54% / 76%

Notes: Alternate names for optix are Dmel and Six3. Alternate names for amos are helix-loop-helix, absent MD neurons, reduced olfactory organs, and rough eye. Worniu also matches many zinc finger transcription factors with lower similarity.

## DISCUSSION

We cannot claim to have identified every gene transcript in Müller’s organ whose abundance was changed by noise exposure, but genes participating in most major functions were probably found. We might have failed to identify very low abundance transcripts, but we saw abundance values over almost 5 log units and in all the expected major functional groups.

### Abundance Ratio Distributions and the Significance of Ratio Measurements

Changes in gene transcript abundance provide an important window into processes such as cancer development, aging, drug therapies, sensory stimulation, etc. and are encouraged by the increasing quality and availability of transcriptome data. But how significant are measured abundance ratios, compared to the experimental variability? A review of approaches to abundance ratio analysis, primarily for human cancer work, pointed out that reads are not often uniformly distributed along transcripts, and that total transcriptome reads from each gene provide an important, often ignored measure (28).

We based our approach on the previous finding that counting all the reads matching the coding frame gave relative abundance values that agreed with quantitative PCR measurements (26). The Cauchy distribution (27) can arise from the ratio of two normally distributed random variables with zero means. In the current situation, we had only single measurements of each abundance (control and noise exposed), but it is reasonable to assume that many independently made transcriptomes, each with multiple steps between tissue and final sequencing, would produce normally distributed abundance values for each transcript. This issue is worth exploring when multiple repeated transcriptomes become more feasible.

The shape of the Cauchy distribution implies that relatively large changes in gene expression ratios are difficult to interpret. For example, a 50% change might be impressive on a bar graph but would only fall within the expected range of Fig. 2. This could have important consequences for interpreting the increasing amount of transcriptome data encountered in clinical and experimental work.

### Changes in the Mechanical Properties of Müller’s Organ

The two most strongly increased transcripts (Fig. 3, Table 2) encode chemosensory proteins. Although this family is eponymously involved in chemical sensation, they are widespread across tissues and phyla, with a range of functions based on binding to lipids (29). They have also been associated with development and modification of the integument (30). This agrees with the finding that four of the other increased transcripts encode structural glycoproteins that are used to construct the endocuticle layer of the integument. Increased expression of homologous transcripts in different termite castes was associated with increased thickness and hardness of the endocuticle (31), so noise exposure probably caused a thicker, harder tympanum. Another increased transcript was a lysozyme-like. These enzymes break glycosidic bonds, including those in chitin, supporting the picture of cuticle remodeling. However, apolipophorins are also involved in cuticular construction (32) and some of these were substantially reduced (Fig. 3).

Insect ears, like human ears, use active movement to improve sensitivity (6, 7). Our previous study found that noise exposure caused increased displacement of the tympanum by sound (3) and suggested three possible causes for this based on active and passive components of the sensilla, plus possible muscle attachments. We must now add changes in the cuticle of the tympanum, or possibly its supporting structures. Solitary locusts (8) had larger tympanal movement over a wide frequency range, but stronger neural responses only to high frequencies (15–20 kHz), whereas aged locusts had lower neural responses that were not correlated with tympanal movement (33), so the relationship between tympanal movement and sensory response is not straightforward. Aged *Drosophila* ears had less mechanical gain and reduced stiffness, which was used to predict a 50% reduction in functioning mechanically activated ion channels (7). However, no changes in the passive mechanical structures were recorded.

Two muscle protein transcripts, troponin and myosin heavy chain, were reduced by noise exposure (Fig. 3, Table 3). Myosin light chain was also reduced but slightly less. Therefore, reduced muscle tension is another candidate for increased tympanal compliance. Although a softer tympanum might be expected to move more easily, a stiffer tympanum might resist flexion between different regions of the cuticle, leading to greater movement at Müller’s organ. Resolution of these issues could be helped by recording from or manipulation of tympanal muscle to determine its contributions to passive and active tympanal movement. A better understanding of the frequency dependent mechanical properties of the complex tympanal structure is also desirable (22).

### Metabolic Consequences of Noise Exposure

The most strongly reduced transcripts (Fig. 3) were from genes associated with cellular metabolism (vitellogenins, apolipophorin, gamma butyrobetaine dioxygenase, pancreatic lipase-related protein, and hexamerins), as well as enzymes that could support a wide range of cellular processes (carboxylesterase, greglin, GILT-like, and prostatic acid phosphatase). Neurons are active cells, particularly because action potentials consume considerable energy (34). Stimulation with a loud sound for 24 h presumably generated many action potentials in Müller’s organ. Noise exposure also produced metabolic stress in *Drosophila* auditory neurons (2), including changes in mitochondrial structure.

Turnover of mRNA transcripts is a complex process (35). Transcripts have half-lives ranging from a few minutes to many hours, and numerous mechanisms have been identified that degrade and modify mRNA. Stressed cells are known to reduce general protein synthesis, including aggregation of mRNA into granules targeted for storage or degradation. The duration of noise stimulation here was clearly adequate to initiate or interact with some of these processes. However, it is impossible to tell from the current evidence whether the broad reduction in transcripts supporting cell metabolism reflects feedback processes to protect the tissue from overstimulation, or exhaustion of the cell’s energy production mechanisms. Similarly, we do not yet know if the reduced metabolic capacity caused changes in auditory functions, such as ionic concentrations or muscle contractility.

### Sensory Receptor Currents in Müller’s Organ

Previous experiments found normal membrane electrophysiology in noise-exposed sensory neurons, including the action potentials produced by sound or electrical stimulation (3). However, receptor current was significantly reduced. This suggests that sound exposure does not change general ionic concentrations but does affect mechanically activated ion channels or the transepithelial ion and voltage gradients (19) that drive the current. No reduction was seen in any of the transcripts from seven putative mechanically activated ion channels, confirming previous data (3). That leaves reduced transepithelial gradients as a possibility.

Insect epithelial ion transport involves several ion pumps, exchangers, and channels but is incompletely characterized (20). Although we found 32 transcripts of ion transporters and pumps, plus 24 ion channels, the only change caused by noise exposure was the increased abundance of one Na^+^/H^+^ exchanger (Fig. 3, Table 2). This sequence matches insect genes identified as exchanger 9B2, possibly of mitochondrial origin. However, all those sequences were predicted by genomic transcriptions, without any functions or tissue location.

The present data support our previous suggestion of reduced transepithelial gradients (3) but indicate that it arises indirectly from a loss of transcripts responsible for general cellular energy production. Reduction in noise-exposed *Drosophila* auditory receptor potential was also attributed to reduced metabolic capacity (2).

Two additional neural transcripts were reduced by noise exposure. Clavesins are Golgi apparatus proteins involved in vesicular trafficking (36). This may have been reduced by the overall metabolic effect. Timeless (37) is a component of the circadian mechanism but also involved in DNA replication and repair. Noise stimulation for 24 h probably disrupted circadian maintenance of this transcript.

### What Drives the Noise-Induced Transcriptional Changes?

Transcription factors (TF) were implicated in an insect model of aged deafness (7), and mammalian TFs rescued insect hearing (38). The lack of TFs in the lists of affected transcripts is surprising. Orthopteran TFs are not well described. A review of insect TFs (39) listed only three in *Locusta* and two in *Schistocerca*, compared to 117 in *Drosophila*. Four TFs were identified as age-related regulators of auditory transduction in *Drosophila* (7), but no locust transcripts matching these (Table 4) were affected by noise exposure. The most strongly affected TF was SCH_0487 (1.526 noise/control). This putatively encodes lipopolysaccharide-induced tumor necrosis factor alpha, which has many possible functions, including in lyzosymes, so this may be linked to the increase in the lysozyme-like transcript.

### Models of Deafness

Age and sound exposure can both cause deafness in mammals and insects, but the underlying mechanisms may be different. Age affects more physiological processes, and probably gene transcripts, than sound alone, making interactions between different systems possible. However, the limited data suggest that both mechanical coupling of sound and transduction of receptor current are usually involved. The recent description of the complete *Schistocerca* genome (40) promises to allow more structured studies of this interesting model of deafness.

## GRANTS

This work is supported by the Natural Sciences and Engineering Council of Canada Grant RGPIN/03712 to A. S. French and Leverhulme Trust Early Career Fellowship and a Wellcome Trust Institutional Strategic Support Fund Fellowship awarded to B. Warren. B. Warren was also supported by the Department of Neuroscience, Psychology and Behavior at the University of Leicester.

## DISCLOSURES

No conflicts of interest, financial or otherwise, are declared by the authors.

## AUTHOR CONTRIBUTIONS

A.S.F. and B.W. conceived and designed research; B.W. performed experiments; A.S.F. analyzed data; A.S.F. and B.W. interpreted results of experiments; A.S.F. prepared figures; A.S.F. drafted manuscript; A.S.F. and B.W. edited and revised manuscript; A.S.F. and B.W. approved final version of manuscript.
